# Characterization of UDP-Activated Purinergic Receptor P2Y_6_ Involved in Japanese Flounder *Paralichthys olivaceus* Innate Immunity

**DOI:** 10.3390/ijms19072095

**Published:** 2018-07-19

**Authors:** Shuo Li, Jiafang Li, Nan Wang, Gaixiang Hao, Jinsheng Sun

**Affiliations:** Tianjin Key Laboratory of Animal and Plant Resistance, College of Life Sciences, Tianjin Normal University, Tianjin 300387, China; skyls@tjnu.edu.cn (S.L.); 13012237391@163.com (J.L.); m15620533237@163.com (N.W.); 18234099132@163.com (G.H.)

**Keywords:** P2Y_6_ receptor, purinergic signaling, innate immunity, teleost fish, *Paralichthys olivaceus*

## Abstract

Uridine 5’-diphosphate (UDP)-activated purinergic receptor P2Y_6_ is a member of a G-protein-coupled purinergic receptor family that plays an important role in mammalian innate immunity. However, the role of the P2Y_6_ receptor (P2Y_6_R) in fish immunity has not been investigated. In this report, we characterized a *P2Y_6_R* gene from Japanese flounder (*Paralichthys olivaceus*) and examined its role in fish innate immunity. Sequence analysis reveals that the Japanese flounder P2Y_6_R protein is conserved and possesses four potential glycosylation sites. Quantitative real-time RT-PCR analysis shows that *P2Y_6_R* is broadly distributed in all examined Japanese flounder tissues with dominant expression in the liver. In addition, *P2Y_6_R* gene expression was up-regulated in head kidney macrophages (HKMs) upon lipopolysaccharides (LPS) and poly(I:C) stimulations but down-regulated by LPS challenge in peripheral blood leukocytes (PBLs). Furthermore, pharmacological inhibition of the endogenous P2Y_6_ receptor activity by the potently selective P2Y_6_R antagonist, MRS 2578, greatly up-regulated pro-inflammatory cytokine *interleukin (IL)-1β*, *IL-6* and *TNF-α* gene expression in PBL cells treated with UDP. Moreover, LPS- and poly(I:C)-induced gene expression of *IL-1β* and *TNF-α* in Japanese flounder PBL cells was attenuated significantly by inhibition of P2Y_6_R activity with antagonist MRS 2578. Collectively, we, for the first time, showed the involvement of functional purinergic P2Y_6_R in fish innate immunity.

## 1. Introduction

Nucleotides, such as adenosine 5′-triphosphate (ATP), adenosine 5′-diphosphate (ADP), uridine 5′-triphosphate (UTP), and uridine 5′-diphosphate (UDP), perform important roles in modulating inflammatory and cell death responses following their release into the extracellular milieu and activation of purinergic receptors at the plasma membrane surface [1,2,3]. Purinergic receptors include three different families: P2Y, P2X, and P1 receptors, which are widely expressed in all mammalian cell types. Mammalian cells can express various combinations of the eight known subtypes of P2Y receptor (P2Y_1_R, P2Y_2_R, P2Y_4_R, P2Y_6_R, and P2Y_11_R–P2Y_14_R) that belong to the G-protein-coupled receptor (GPCR) superfamily, the seven P2X receptors (P2X1R–P2X7R) that act as ATP-gated ion channels and the four different G-protein-coupled adenosine (P1) receptors [4]. 

P2Y receptors show marked differences in ligand selectivity and specificity of G protein coupling [5]. Unlike other P2 receptors, P2Y_6_R is selectively activated by nucleotide UDP but not by ATP [6]. P2Y_6_R was initially identified as an immune mediator of microglial phagocytosis [7]. Subsequently, P2Y_6_R has received much more attention for its important role in adjusting immune cell functions. It has been demonstrated that UDP and lipopolysaccharides (LPS)-induced IL-8 release from human monocytic THP-1 cells is mediated by an autocrine stimulation of the P2Y_6_ receptor [8]. P2Y_6_ receptors also significantly up-regulated the mRNA levels of *IL-8*, *IP-10*, and *IL-1β* in human monocytic cells stimulated with UDP [9]. In addition, activation of the P2Y_6_ receptor by UDP can increase osteoclasts’ survival through activation of NF-κB [10]. Furthermore, studies also demonstrated that the P2Y_6_ receptor is a novel mediator in up-regulating innate immune response against invading pathogens through recruiting monocytes/macrophages [11].

We previously showed that functional P2Y_2_ and P2Y_12_ receptors are expressed in Japanese flounder immune cells [12], while the presence and immune function of other P2YRs in fish is still unknown. In this report, we show that in addition to previously demonstrated P2Y_2_ and P2Y_12_ receptors, Japanese flounder immune cells also express functional P2Y_6_ receptors. Using pharmacological approaches, we further reveal the association of the P2Y_6_ receptor with TLR3/4-mediated immune signaling in fish.

## 2. Results and Discussion

### 2.1. Sequence Analysis of Japanese Flounder P2Y_6_ Receptor

**Figure 1:**
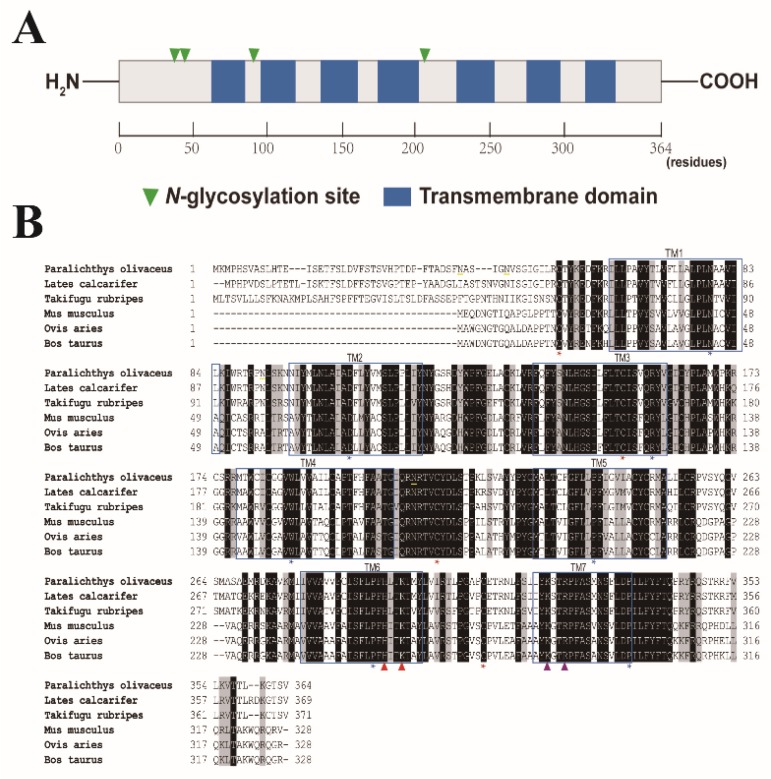
Schematic domain structure and sequence alignment of P2Y_6_ receptor proteins. (**A**) The schematic domain structure of Japanese flounder P2Y_6_ receptor protein. A scale bar of the amino acid residues is indicated at the bottom. (**B**) Alignment of the amino acid sequences of P2Y_6_ receptor proteins from different species. The amino acid sequence of Japanese flounder *Paralichthys olivaceus* P2Y_6_ receptor protein (XP_019943561.1) is compared with its orthologs from: *Lates calcarifer* (XP_018543417.1); *Takifugu rubripes* (XP_003977127.2); *Mus musculus* (NP_898991.1); *Ovis aries* (XP_014956561.1); and, *Bos taurus* (NP_001179224.1). The transmembrane domains (TM1-TM7) are boxed. The potential *N*-linked glycosylation sites in Japanese flounder P2Y_6_ receptor protein are underlined in yellow. The four cysteine residues involved in disulfide bridges and the conserved residues among G-protein-coupled receptors of each transmembrane domain are denoted with red and blue stars, respectively. The conserved positive-charged residues responsible for nucleotide binding in the H-X-X-R/K motif and the Y-Q/K-X-X-R motif among P2Y_6_ receptors are indicated by red and purple triangles, respectively. Identity is denoted by shaded white letters, and similarity is shown by shaded black letters.

**Figure 2:**
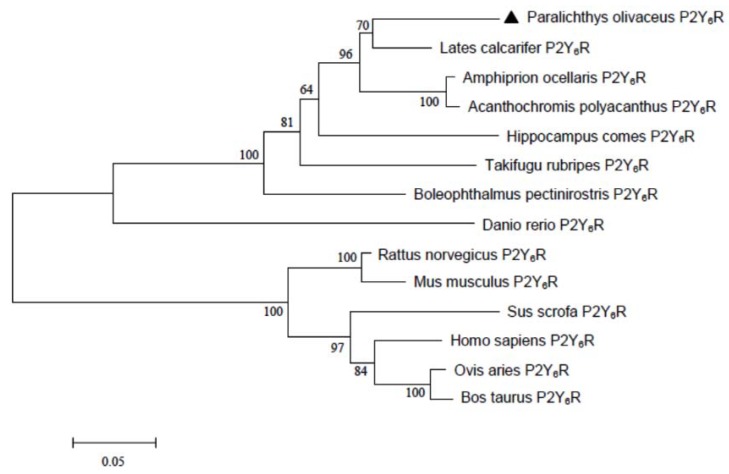
Phylogenetic relationship analysis of Japanese flounder P2Y_6_ receptor and its counterparts from selected mammalian and fish species. Protein sequences were aligned with the ClustalW program and the maximum-likelihood phylogenetic tree was constructed using MEGA software version 5.0 with default parameters. GenBank accession numbers are: *Danio rerio* P2Y_6_R (XP_694459.2); *Amphiprion ocellaris* P2Y_6_R (XP_023144707.1); *Acanthochromis polyacanthus* P2Y_6_R (XP_022062059.1); *Boleophthalmus pectinirostris* P2Y_6_R (XP_020796203.1); *Hippocampus comes* P2Y_6_R (XP_019741395.1); *Rattus norvegicus* P2Y_6_R (NP_476465.1); *Sus scrofa* P2Y_6_R (NP_001231225.2); *Homo sapiens* P2Y_6_R (AAB80713.1); and the list in the legend of Figure 1.

Japanese flounder P2Y_6_R protein comprises 364 amino acid residues with a calculated molecular mass of 41.3 kDa and an isoelectric point of 9.52. A Basic Local Alignment Search Tool (BLAST) search of the National Center for Biotechnology Information (NCBI) database reveals that the P2Y_6_ receptor is highly conserved (i.e., greater than 80% sequence identity among different teleost species). Sequence analysis revealed that Japanese flounder P2Y_6_R harbors seven transmembrane domains (TM1: ^62^Ile-^84^Leu; TM2: ^97^Asn-^119^Tyr; TM3: ^139^Phe-^161^Val; TM4: ^178^Met-^204^Gly; TM5: ^229^Met-^251^Ala; TM6: ^279^Ile-^299^Tyr; and TM7: ^319^Ile-^335^Pro) with an extracellular amino terminus and an intracellular carboxyl terminus (Figure 1). In addition, four cysteine residues (^53^Cys, ^154^Cys, ^212^Cys, and ^310^Cys) involved in disulfide bridges and the conserved residues (^79^Asn, ^107^Asp, ^159^Arg, ^187^Trp, ^239^Pro, ^291^Pro, and ^335^Pro) among GPCRs of each transmembrane domain [13] are also preserved among the fish and mammalian P2Y_6_Rs (Figure 1B), suggesting that these essential structures for P2Y_6_Rs remains through evolution. Furthermore, Japanese flounder P2Y_6_R possesses four potential glycosylation sites (^39^Asn, ^44^Asn, ^92^Asn, and ^208^Asn) and several phosphorylation sites which are involved in receptor desensitization and internalization [14]. Finally, there are two consensus motifs, including a H-X-X-R/K motif in TM6 and a Y-Q/K-X-X-R motif in TM7, in Japanese flounder P2Y_6_R, in which the positively charged residues that may interact with the negative charges of the phosphate groups of nucleotides [15] are also conserved. However, Japanese flounder P2Y_6_R shares only 32% sequence identity to the counterpart from human beings. Phylogenetic analysis further revealed that fish P2Y_6_R proteins were clustered into a separated clade that is distinct from the clade formed by mammalian P2Y_6_R proteins (Figure 2), indicating that fish P2Y_6_R proteins are diverged from mammalian P2Y_6_R proteins through evolution.

### 2.2. Expression of P2Y_6_ Receptor mRNA Transcripts in Japanese Flounder Tissues

**Figure 3:**
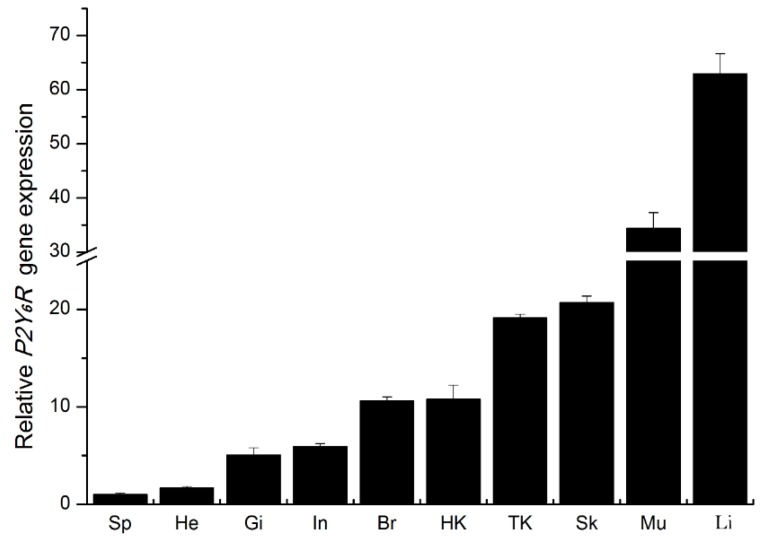
Quantitative real-time RT-PCR analysis of basal expression of *P2Y_6_* receptor mRNA transcripts in Japanese flounder tissues. Individual tissues from five healthy Japanese flounders were equally pooled for quantification of the relative gene expression level of *P2Y_6_R* with *β-actin* as an internal reference gene. Abbreviations for x-axis: Sp: spleen; He: heart; Gi: gill; In: intestine; Br: brain; HK: head kidney; TK: trunk kidney; Sk: skin; Mu: muscle; Li: liver. Data represent means ± standard deviations from triplicate experiments.

The P2Y_6_ receptor shows a ubiquitous distribution in mammalian tissues. In human beings, *P2Y_6_* receptor mRNA transcripts were detected at different levels in all 20 tissues with high levels in those of the spleen, the placenta and the kidney [16]. *P2Y_6_R* mRNA was also abundantly expressed in various rat tissues including the lung, the stomach, the intestine, the spleen, the mesentery, the heart, and most prominently in the aorta [17]. Although currently there are no data reporting the tissue expression of *P2Y_6_R* in teleost, quantitative real-time RT-PCR (RT-qPCR) analysis revealed a wide but unequal tissue distribution of *P2Y_6_R* mRNA in Japanese flounder. In particular, dominant expression of Japanese flounder *P2Y_6_R* mRNA transcripts was found in the liver while the least expression was in the spleen (Figure 3). We have shown previously the transcriptional presence of *P2X2*, *P2X4*, *P2X7*, *P2Y_2_*, and *P2Y_12_* receptors in various Japanese flounder tissues [12,18,19,20]. The diversity of purinergic receptor subtypes present in Japanese flounder tissues and cells, addresses an essential role of purinergic receptors in fish through triggering different downstream signaling events.

### 2.3. The Effects of Inflammatory Challenge on P2Y_6_ Receptor Expression in Japanese Flounder Immune Cells

**Figure 4:**
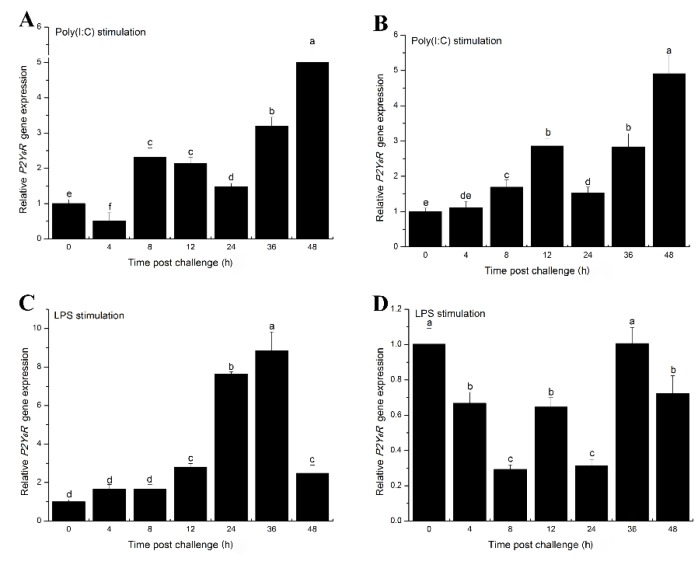
*P2Y_6_* receptor gene expression after inflammatory challenges in Japanese flounder immune cells. Expression of *P2Y_6_* receptor mRNA in response to poly(I:C) (**A**) and (**B**) and lipopolysaccharides (LPS) (**C**) and (**D**) stimulations in Japanese flounder head kidney macrophages (**A**) and (**C**) and peripheral blood leukocytes (**B**) and (**D**) relative to housekeeping gene *β-actin* was determined by quantitative real-time PCR. The expression of *P2Y_6_R* in response to inflammatory challenges was normalized to untreated control cells (set to 1). Data are presented as means ± standard deviations (*n* = 3). Groups marked by different lowercase letters above each bar represent a significant difference at *p* < 0.05. The same letters indicate no significant difference between groups.

Previous studies demonstrated that inflammation can increase expression of the P2Y_6_ receptor in epithelial cells [21,22]. We therefore examined expression of the Japanese flounder *P2Y_6_* receptor in response to inflammatory challenges. Using quantitative real-time RT-PCR, we found that *P2Y_6_* receptor mRNA transcripts were significantly induced in head kidney macrophage (HKM) cells following stimulation with poly(I:C) or LPS (Figure 4A,C, respectively). Similar to our findings, robust induction of *P2Y_6_R* by LPS treatment was also observed in mouse heart and kidney tissues [23]. In addition, *P2Y_6_* receptor expression was increased in peripheral blood leukocyte (PBL) cells after poly(I:C) treatment (Figure 4B). In contrast, LPS down-regulated *P2Y_6_* receptor expression in PBL cells (Figure 4D). Although *P2Y_6_* receptors are transcriptionally modulated by inflammatory stimulation, the transcriptional mechanism remains unknown. The up-regulated *P2Y_6_* receptor may function as a sensor for inflammatory stimuli by sensing the released UDP signals in Japanese flounder immune cells. The *P2Y_6_* receptor was induced by LPS in HKM cells but down-regulated in PBL cells, probably reflecting a yet unidentified cell-type dependent mechanism. 

### 2.4. P2Y_6_ Receptor-Mediated Innate Immune Response in Japanese Flounder PBL Cells

**Figure 5:**
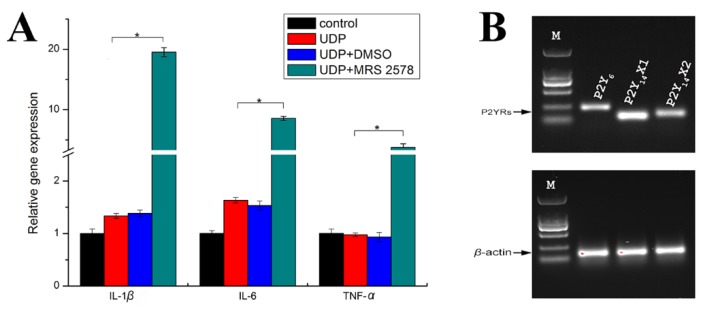
P2Y_6_ receptor mediates pro-inflammatory cytokine gene expression in Japanese flounder PBL cells stimulated with uridine 5′-diphosphate (UDP). (**A**) Inhibition of P2Y_6_ receptors activity affects *IL-1β*, *IL-6* and *TNF-α* gene expression in Japanese flounder PBL cells stimulated with UDP. Japanese flounder PBL cells were pre-incubated with or without 10 µM MRS 2578 for 30 min and then stimulated with 50 µM UDP for 2 h in the presence of 10 µM MRS 2578 or same volume of vehicle dimethyl sulfoxide (DMSO). The relative pro-inflammatory cytokine gene expression levels were examined by quantitative real-time RT-PCR and were normalized to untreated control cells (set to 1). Significant difference between UDP treated groups and UDP plus MRS 2578 treated groups was determined by the Student’s *t*-test and is indicated by brackets and asterisks at *p* < 0.05. Addition of DMSO did not affect the UDP-induced pro-inflammatory cytokine gene expression. Data are presented as means ± standard deviations of triplicate determinants from one representative experiment. Similar results were obtained in two other separated experiments. (**B**) Reverse transcription PCR (RT-PCR) analysis of *P2Y_6_* and *P2Y_14_* receptor variants (*P2Y_14_X1* and *P2Y_14_X2*) gene expression in Japanese flounder PBL cells. The presence of *P2Y_6_* and *P2Y_14_* receptor mRNA transcripts in Japanese flounder PBL cells was analyzed by RT-PCR with *β-actin* served as a loading control.

Innate immune response is a critical step in the fish defense system against infectious agents. To explore the potential role of the P2Y_6_ receptor in Japanese flounder innate immunity, the endogenous P2Y_6_R activity in Japanese flounder PBL cells were pharmacologically inhibited by pre-incubation with the selective P2Y_6_ receptor antagonist, *N*,*N*″-1,4-Butanediylbis(*N*′-(3-isothiocyanatophenyl))thiourea (MRS 2578) [7,24], and the resultant consequence on pro-inflammatory cytokine *IL-1β*, *IL-6*, and *TNF-α* mRNA production was examined. As shown in Figure 5A, UDP treatment slightly up-regulated *IL-1β* and *IL-6* expression but did not affect *TNF-α* expression compared with untreated control cells, while inhibition of P2Y_6_ receptor activity by MRS 2578 significantly increased all gene expression, suggesting that activation of P2Y_6_R by UDP may suppress pro-inflammatory gene expression. Vehicle dimethyl sulfoxide (DMSO, a substance used to dissolve P2Y_6_R antagonist MRS 2578) plus UDP treated groups did not show any significant difference in pro-inflammatory gene expression compared with UDP-only treated groups, ruling out the effects of DMSO on target gene expression. Similar to our observation, UDP treatment can also up-regulate *IL-1β* but not *TNF-α* gene expression in human promonocytic U937 cells [9]. Because UDP can activate not only P2Y_6_R but also P2Y_14_R [13], this unchanged *TNF-α* gene expression in PBL cells may be due to the balanced actions of P2Y_6_ and P2Y_14_ receptors. This hypothesis was supported by the presence of two *P2Y_14_* receptor variant mRNA transcripts (*P2Y_14_RX1* and *P2Y_14_RX2*) in PBL cells (Figure 5B). 

**Figure 6:**
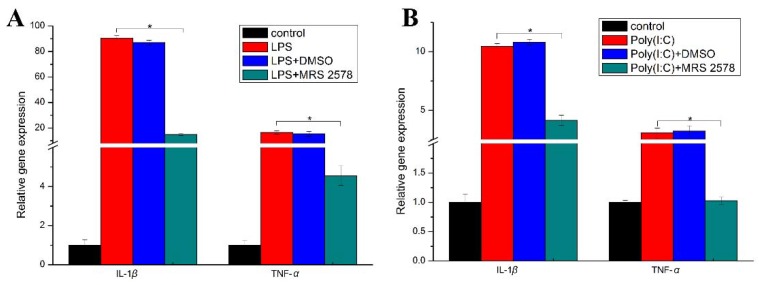
Effects of P2Y_6_ receptor antagonist MRS 2578 on pathogen-associated molecular pattern (PAMP)-induced pro-inflammatory cytokine gene expression in Japanese flounder PBL cells. Japanese flounder PBL cells were pre-incubated with 10 µM MRS 2578 for 30 min and then stimulated with 20 µg/mL LPS (**A**) or poly(I:C) (**B**) for 2 h in the presence of 10 µM MRS 2578 or same volume of vehicle DMSO. The relative gene expression level of *IL-1β* and *TNF-α* was determined by quantitative real-time RT-PCR and normalized to untreated control cells (set to 1). Data are presented as means ± standard deviations of triplicate determinants from one representative experiment. Similar results were obtained in two other separated experiments. Significance between PAMP and PAMP plus MRS 2578 treated groups is determined by the Student’s *t*-test and is indicated by brackets and asterisks at *p* < 0.05.

The involvement of P2Y_6_ receptor in TLR-mediated immune signaling was documented in human monocytic cells [8,25,26] and mouse macrophages [27]. We next examined the potential role of P2Y_6_R in pathogen-associated molecular patterns (PAMPs)-induced innate immunity in Japanese flounder PBL cells. As shown in Figure 6, gene expression of pro-inflammatory cytokines *IL-1beta* and *TNF-α* was significantly induced when Japanese flounder PBL cells were treated with TLR3 and 4 ligands, poly(I:C) and LPS, respectively. However, the elevated expression was reduced significantly by addition of selective P2Y_6_R pharmacological antagonist MRS 2578. A recent study reported augmented pro-inflammatory responses in mammalian macrophages by the P2Y_6_ receptor [28]. Thus, the P2Y_6_ receptor may positively regulate PAMP-induced inflammatory response in Japanese flounder immune cells. In addition, these findings also suggest that there is potential crosstalk between P2Y_6_R-mediated purinergic signaling and TLR-mediated immune signaling in fish.

In conclusion, the present studies for the first time reveal the involvement of functional purinergic receptor P2Y_6_ in Japanese flounder innate immunity.

## 3. Material and Methods 

### 3.1. Ethics Statement

All experiments were conducted in accordance with NIH guidelines for the care and use of experimental animals and these studies were specifically approved by the Ethics Committee of College of Life Sciences, Tianjin Normal University (#2018-01, 9-January-2018).

### 3.2. Fish Maintenance 

Japanese flounder *P. olivaceus* from a local fish farm in Tianjin, China, were cultured in an aerated recirculating seawater system in the laboratory at 21 °C for two weeks before experimentations. Fish were clinically examined before experimentation and only healthy animals without any pathological signs were selected for use in experiments. 

### 3.3. RNA Purification, cDNA Synthesis and Gene Cloning 

Total RNA from the liver tissue of Japanese flounder was isolated using TRIzol reagent (Invitrogen, Carlsbad, CA, USA) and was treated with RNase-free DNase I (Invitrogen) to eliminate genomic DNA contamination before transcription. The treated RNA was then reverse transcribed into cDNAs using a Superscript III cDNA synthesis kit (Invitrogen) following the manufacturer’s directions. Japanese flounder P2Y_6_ receptor cDNA sequence (XM_020088001) was retrieved from the NCBI database and cloned by reverse transcription PCR with the primers listed in Table 1 using cDNAs from liver tissue as templates. DNase I-treated samples did not amplify any PCR products, confirming that there is no genomic DNA contamination.

### 3.4. Sequence Alignment and Phylogenetic Analysis 

Multiple sequence alignments of the amino acid sequences of Japanese flounder P2Y_6_ receptor and its counterparts in other selected vertebrate species were conducted using ClustalW program (http://www.ebi.ac.uk/clustalw/) [29]. The maximum-likelihood phylogenetic tree of selected P2Y_6_ receptors was constructed using MEGA software (version 5.0) with 1000 bootstrap replications. 

### 3.5. Analysis of P2Y_6_ Receptor Gene Expression in Japanese Flounder Tissues 

The tissue expression profile of *P2Y_6_* mRNA transcripts in healthy juvenile *P. olivaceus* was analyzed by quantitative real-time RT-PCR (see Section 3.8). Japanese flounder tissues including those of the brain, the gill, the head kidney, the trunk kidney, the heart, the liver, the skin, the muscle, the intestine, and the spleen from five healthy animals (average 500 ± 20 g) were aseptically separated, collected, and pooled. Total RNA was purified and treated with DNase I as described previously. Aliquots (2 µg) of total RNA after DNase I treatment from each type of tissue were transcribed into cDNAs in a 20 µL reaction mixture using SuperScript III ribonuclease H^−^ reverse transcriptase. 

### 3.6. Isolation, Cell Culture and Inflammatory Stimulation of Japanese Flounder Head Kidney Macrophages and Peripheral Blood Leukocytes

Japanese flounder HKMs and PBLs were prepared by discontinuous Percoll density gradient centrifugation (1.020/1.070 and 1.070/1.077, respectively; GE Biosciences, Chicago, IL, USA). After centrifugation at 3000 rpm for 30 min at 4 °C, the white interface between the lower and upper layers was collected and washed 3 times with cold phosphate buffer saline (PBS). Freshly prepared HKMs and PBLs were re-suspended in a culture medium (RPMI 1640 supplemented with 10% fetal bovine serum (FBS) and 1% penicillin–streptomycin liquid) and grown at 21 °C.

After overnight culture, HKMs and PBLs (5 × 10^6^ cells/well in a 24-well plate; ThermoFisher Scientific, Suzhou, China) were incubated with 20 µg/mL LPS or poly(I:C) in a culture medium in the absence of FBS for 0, 4, 8, 12, 24, 36, and 48 h. After treatment, total RNA was purified from the cells and reverse transcribed into cDNAs. Gene expression changes of the *P2Y_6_* receptor following PAMP treatment were evaluated by quantitative real-time RT-PCR as described below.

### 3.7. Pharmacological Treatment

To explore the role of P2Y_6_ receptors in Japanese flounder innate immunity, Japanese flounder PBL cells were pre-incubated with or without 10 µM selective P2Y_6_ receptor antagonist *N*,*N*″-1,4-Butanediylbis(*N*′-(3-isothiocyanatophenyl))thiourea (MRS 2578; Tocris Bioscience, Minneapolis, MN, USA) [7,24] for 30 min to inhibit endogenous P2Y_6_R activity, prior to treatment with 20 µg/mL LPS or poly(I:C), or 50 µM (optimized concentration) UDP (an endogenous agonist for activation of P2Y_6_R; Sigma-Aldrich, St. Louis, MO, USA) for 2 h in the presence or absence of antagonist MRS 2578. After treatments, the resultant consequence on pro-inflammatory cytokine *IL-1β*, *IL-6*, and *TNF-α* mRNA production was assessed by quantitative real-time RT-PCR. In a parallel experiment, to exclude the effects of DMSO on pro-inflammatory cytokine gene expression, cells were also treated with LPS, poly(I:C), or UDP in the presence of vehicle DMSO, which was used to dissolve MRS 2578.

### 3.8. Gene Expression Analysis

The relative gene expression levels of *P2Y_6_R* and pro-inflammatory cytokines were quantified by quantitative real-time RT-PCR with the comparative 2^−^^△△*C*t^ quantification method [30]. Quantitative real-time RT-PCR was performed on an Applied Biosystems^®^ 7500 Fast Real-Time PCR System (ThermoFisher Scientific) with each sample in triplicate, using AceQ qPCR SYBR Green Master Mix kit (Vazyme Biotech Co. Ltd., Nanjing, China), following the manufacturer’s instructions. *β-actin* was served as an internal reference gene and the primer sets for detection of the individual genes are summarized in Table 1. The protocol used for quantitative real-time RT-PCR included initial denaturation at 95 °C for 5 min, 40 cycles at 95 °C for 10 s, and 60 °C for 35 s, followed by melting curve analysis (95 °C for 15 s, 60 °C for 60 s, and 95 °C for 15 s), which was detailed in our previous study [31]. 

### 3.9. Statistical Analysis

All data are presented as means ± standard deviations from triplicate experiments. Statistical significance was analyzed by SPSS software (version 11.0). The Student’s *t*-test was applied in two-group analysis. Differences between the means of multiple groups were compared by one-way analysis of variance (ANOVA), followed by Duncan’s multiple range post hoc analysis. A *p* value less than 0.05 was considered statistically significant.

## Figures and Tables

**Table 1:** The sequences of primers used in this study.

Primer Name	Sequences (5′→3′)	Applications
*P2Y_6_*-f	ATGAAGATGCCACATTCT	Gene cloning
*P2Y_6_*-r q*P2Y_6_*-f q*P2Y_6_*-r	TTGTTCGCTCACACACTC AGTGCGGAGATGCGGGAC GGTATCGTGGCTGTGTGAAGTA	quantitative real-time RT-PCR
*IL-1β*-f *IL-1β*-r *IL-6*-f *IL-6*-r	CCTGTCGTTCTGGGCATCAA CACCCCGCTGTCCTGCTT CAGCTGCTGCAAGACATGGA GATGTTGTGCGCCGTCATC	
*TNF-α*-f	CCGACTGGATGTGTAAGGTG	
*TNF-α*-r	GTTGTGGGGTTCTGTTTTCTC	
*β-actin*-f *β-actin*-r	AGGTTCCGTTGTCCCG TGGTTCCTCCAGATAGCAC	

f: forward; r: reverse.
